# Achieving Optimal Growth through Product Feedback Inhibition in Metabolism

**DOI:** 10.1371/journal.pcbi.1000802

**Published:** 2010-06-03

**Authors:** Sidhartha Goyal, Jie Yuan, Thomas Chen, Joshua D. Rabinowitz, Ned S. Wingreen

**Affiliations:** 1 Department of Physics, Princeton University, Princeton, New Jersey, United States of America; 2 Department of Chemistry, Princeton University, Princeton, New Jersey, United States of America; 3 Department of Applied Mathematics, Princeton University, Princeton, New Jersey, United States of America; 4 Department of Molecular Biology, Princeton University, Princeton, New Jersey, United States of America; University of Illinois at Urbana-Champaign, United States of America

## Abstract

Recent evidence suggests that the metabolism of some organisms, such as *Escherichia coli*, is remarkably efficient, producing close to the maximum amount of biomass per unit of nutrient consumed. This observation raises the question of what regulatory mechanisms enable such efficiency. Here, we propose that simple product-feedback inhibition by itself is capable of leading to such optimality. We analyze several representative metabolic modules—starting from a linear pathway and advancing to a bidirectional pathway and metabolic cycle, and finally to integration of two different nutrient inputs. In each case, our mathematical analysis shows that product-feedback inhibition is not only homeostatic but also, with appropriate feedback connections, can minimize futile cycling and optimize fluxes. However, the effectiveness of simple product-feedback inhibition comes at the cost of high levels of some metabolite pools, potentially associated with toxicity and osmotic imbalance. These large metabolite pool sizes can be restricted if feedback inhibition is ultrasensitive. Indeed, the multi-layer regulation of metabolism by control of enzyme expression, enzyme covalent modification, and allostery is expected to result in such ultrasensitive feedbacks. To experimentally test whether the qualitative predictions from our analysis of feedback inhibition apply to metabolic modules beyond linear pathways, we examine the case of nitrogen assimilation in *E. coli*, which involves both nutrient integration and a metabolic cycle. We find that the feedback regulation scheme suggested by our mathematical analysis closely aligns with the actual regulation of the network and is sufficient to explain much of the dynamical behavior of relevant metabolite pool sizes in nutrient-switching experiments.

## Author Summary

Bacteria live in remarkably diverse environments and constantly adapt to changing nutrient conditions. Recent evidence suggests that some bacteria, such as *E. coli*, are extraordinarily efficient in producing biomass under a variety of different nutrient conditions. This observation raises the question of what physical mechanisms enable such efficiency. Here, we propose that simple product-feedback inhibition by itself is capable of leading to such optimality. Product-feedback inhibition is a metabolic regulatory scheme in which an end product inhibits the first dedicated step of the chain of reactions leading to its own synthesis. Our mathematical analysis of several representative metabolic modules suggests that simple feedback inhibition can indeed allow for optimal and efficient biomass production. However, the effectiveness of simple product-feedback inhibition comes at the cost of high levels of some metabolite pools, potentially associated with toxicity and osmotic imbalance. These large metabolite pools can be restricted if feedback inhibition is ultrasensitive. We find that the feedback regulation scheme suggested by our mathematical analysis closely aligns with the actual regulation of the nitrogen assimilation network in *E. coli* and is sufficient to explain much of the dynamical behavior of relevant metabolite pool sizes seen in experiments.

## Introduction

Much is known about the metabolic reactions that lead to the production of biomass and energy in cells. However, understanding the logic of metabolic regulation has been challenging due to the network's scale and complexity. Flux-balance analysis (FBA), a constraint-based computational approach, has been used to show that some microorganisms, including *E. coli*, maximize their growth rates per molecule of carbon source consumed [1]. FBA uses mass conservation to predict optimal growth rates as well as fluxes [2]. In its simplest form, FBA assumes that cells regulate fluxes to produce biomass at the maximum rate possible given a particular limiting input flux. Recently, FBA has been successfully applied to additional microorganisms [3]–[5], and to objective functions other then maximizing biomass [6], e.g. maximization of ATP production [7] or minimization of metabolic adjustment in response to perturbations in metabolic network [8]. Attempts to include regulatory [9], [10], thermodynamic [11], [12], and environment-specific constraints have resulted in insights into the structure of metabolic networks, e.g. the organization of redundant pathways [13], [14]. (For a comprehensive list of FBA achievements see reviews by Kauffman *et al*, 2003 and Lee *et al*, 2006). Despite their predictive strength and wide applicability, FBA-based methods are limited; FBA assumes that fluxes are optimal (thereby assuming perfect regulation) but does not reveal how these optimal fluxes are achieved. This leaves open the question: how can cells achieve nearly optimal fluxes for efficient growth?

Previously, some complex bio-molecular networks have been successfully analyzed and understood in terms of simple modules [15], e.g. the eukaryotic cell cycle [16], [17]. In the same spirit, we address the question of how to achieve optimal growth using several representative modules drawn from real metabolism. In particular we consider four modules, each of which captures an essential feature of the real metabolic network - i) a linear pathway, ii) a bidirectional pathway, iii) a metabolic cycle, and iv) integration of two different nutrient inputs. Linear pathways, in addition to being common, suggest simple rules for achieving optimal growth. In the second module, representing a bidirectional pathway, metabolites are interconverted, albeit at a cost, with the consequent risk of running a futile cycle (e.g., interconversion of fructose-6-phosphate and fructose-1,6-bisphosphate (FBP)). In the third module we analyze a metabolic assimilation cycle. A metabolic cycle can be visualized as a linear pathway where the end product is essential for the first step of the pathway. Two important examples of metabolic cycles are the TCA cycle and the glutamine-glutamate nitrogen-assimilation cycle. Finally, the fourth module addresses the problem of balancing two different inputs, carbon and nitrogen. This module takes into account the ability of microbes to assimilate nitrogen in the form of ammonium via an ATP-independent pathway or a higher affinity ATP-dependent one. When nitrogen is scarce, the ATP-dependent pathway is utilized, whereas when carbon is scarce, it is avoided.

For regulation of these modules we invoke only product-feedback inhibition. Since its discovery in the late 1950's, product-feedback inhibition has become recognized as one of the cornerstones of metabolic regulation [18], [19]. This form of regulation was first hypothesized by Novick and Szilard [20] for the tryptophane biosynthetic pathway from chemostat experiments, and has since been found in almost every biosynthetic pathway [21]. Product-feedback inhibition is a regulatory scheme in which the product of metabolism inhibits its own synthetic pathway. Remarkably, in all four of the modules studied, we find that simple product-feedback inhibition is sufficient to control fluxes so as to enable nearly maximally efficient growth.

To test our understanding of the physiological role of product-feedback inhibition, we compared our simple models to actual regulation of the glutamine-glutamate nitrogen assimilation cycle, including its integration with carbon metabolism. We find important similarities between the product-feedback inhibition scheme that we propose based on general principles and the actual regulatory mechanisms present in *E. coli*.

If, as we will argue, simple product-feedback inhibition is enough to achieve nearly optimal growth, why is real metabolic regulation so complex? Metabolic feedback regulation exists at various levels, such as, control of enzyme mRNA transcription [22], reversible enzyme phosphorylation [23], non-competitive allosteric regulation [24], and competition for enzyme active sites [25]. There are many cases where multiple feedback mechanisms work together, e.g. glutamine synthetase is regulated by a bicyclic cascade of covalent modifications and transcriptionally by the NtrC two-component system [26]. Our mathematical analysis suggests that simple feedback regulation, while adequate for flux control, could lead to large metabolite pools, and that accumulation of these pools may be prevented by multiple regulatory mechanisms working in concert to produce ultrasensitive feedback.

## Results

### Models

#### Linear pathway: minimal model

**Figure 1:**
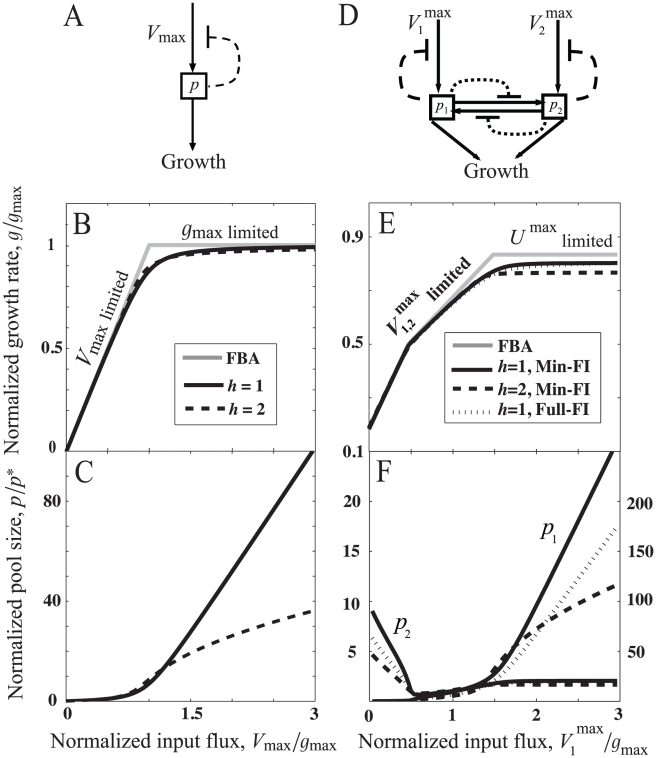
Analysis of metabolic modules: (A) minimal linear pathway and (D) bidirectional pathway, two different regulation schemes are considered – Min-FI scheme: feedbacks only on the input nutrient fluxes (dashed lines), and Full-FI scheme: feedbacks on all the fluxes. (B,C) Results for linear pathway from Eq. 3: (B) 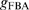, the optimal growth rate given by flux-balance analysis (FBA) (gray curve), and growth rate as a function of 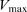 (solid and dashed curves). (C) Metabolite pool size 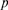 as a function of 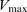. The parameters for numerical solutions are 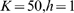 (solid curves) and 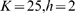 (dashed curves). (E,F) Results for bidirectional pathway from Eq. 5: (E) 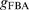 (gray curve), and growth rate as a function of 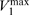 (solid, dotted, and dashed curves). (F) Metabolite pool sizes 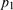 and 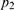 as a function of 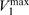. The parameters for FBA and numerical solutions: the maximum input flux, 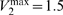, the maximum interconversion flux, 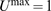, for the Min-FI scheme, 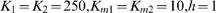 (solid curves) and 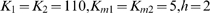 (dashed curves), and for the Full-FI scheme, 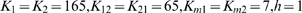 (dotted curves).

To elucidate the main findings of our mathematical analysis, we first consider a minimal metabolic circuit (Fig. 1A) in which an input flux of magnitude 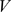 leads to growth at rate 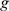 via one metabolite with pool size 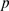. This analysis, while somewhat redundant with prior careful treatments of linear pathways [27], [28], lays out the nomenclature and logic that will be used subsequently for the other modules, where the conclusions are less immediately apparent. For our purpose, we include no intermediates in the linear pathway. The lack of intermediates in the pathway is equivalent to one of the steps of the linear pathway being rate limiting for product formation. In general, input fluxes are limited by nutrient availability, transport, and catabolism, all lumped here into an inequality constraint 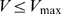. This is an unbranched pathway and thus, at steady-state and assuming no futile cycling, the input flux should equal the efflux leading to growth (e.g., the pathway could make an amino acid, with the efflux being its consumption by protein synthesis leading to growth). The input flux and efflux should both accordingly be proportional to growth rate. Assuming all other components required for growth are freely available, the optimal flux-balance growth rate would be set by the maximum input flux 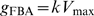, where 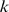 reflects the stoichiometry between the input flux and growth rate. As 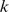 is merely a scaling factor, in all future equations we set it to 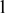 for simplicity. When the maximum input flux become sufficiently high, then growth rate becomes limited by other factors (e.g. other factors in growth medium used to culture cells), never exceeding some maximum 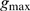. Thus, the optimal flux-balance growth increases linearly with the input flux until it reaches the maximum growth rate 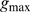 (gray curve in Fig. 1B). In general, to calculate the FBA growth rate one maximizes the steady-state growth rate consistent with the stoichiometric and linear constraints on the various input, output, and internal fluxes.

To go beyond FBA and explicitly consider the regulation of fluxes, we assume product-feedback inhibition acts on the input flux such that
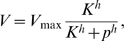
(1)where 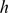 is a Hill coefficient and 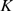 is an inhibition constant. Since the feedback could also be transcriptional, more generally 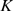 can be interpreted as an effective inhibition constant and 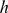 as an effective Hill coefficient. 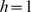 models simple feedback inhibition, while 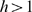 represents ultrasensitive feedback inhibition. Note that Eq. 1 always satisfies the linear constraint 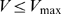.

In our simple linear pathway model, the growth rate 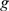 depends exclusively on the size of the metabolite pool 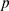. In general, the growth rate 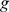 as a function of the pool sizes of 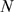 essential metabolites should satisfy the following constraints: 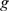 is a monotonically increasing function of each pool, 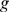 approaches zero if any pool approaches zero, and 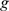 becomes asymptotically independent of each pool 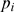 above a certain saturating pool size 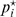. Throughout this work, we use as a growth-rate function
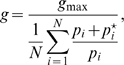
(2)which satisfies the above constraints. This function was obtained as the growth rate of a heteropolymer made from equal stoichiometries of monomers with pool sizes 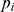 [29]. A pool is called “growth limiting” if 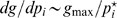.

Combining Eqs. 1 and 2 (with 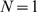) we obtain the kinetic equation for the metabolite pool 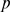,
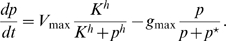
(3)The steady-state metabolite-pool size is obtained by setting the above time derivative to zero, and the growth rate is then calculated using Eq. 2. Intuitively, as long as input flux is limiting for growth (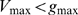), feedback inhibition should be inactive so that there is no reduction of the flow of nutrients into the cell. Therefore, the feedback-inhibition system should be designed such that the feedback remains minimal until 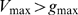, *i.e.* until the ability to produce metabolite 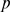 exceeds demand for it. This design can be achieved by choosing parameters in Eq. 3 such that a much larger metabolite pool is required for significant feedback inhibition than is required for saturated growth, that is by choosing 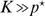. Indeed, the growth rate approaches its optimum as the feedback-inhibition constant 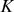 increases (see Text S1). As expected, a large feedback-inhibition constant, 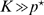, is advantageous for maximizing production of 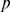 and thus growth rate in the regime where metabolite 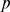 is growth-limiting.

However, there is a trade-off between the growth rate and the metabolite-pool size (Fig. 1C). For non-cooperative feedback (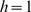), the steady-state pool size is given by
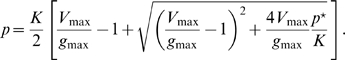
(4)In the asymptotic limit of small input flux, 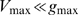 (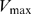-limited regime), the resulting pool size is small, 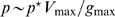, but in the asymptotic limit of large input flux, 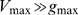 (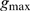-limited regime), the pool size becomes large, 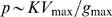, and continues to grow with increasing 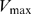. Importantly, in this 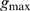-limited regime, the pool size is proportional to the feedback-inhibition constant 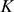. Therefore, while large values of 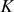 yield nearly optimal growth rates, they also lead to very high metabolite-pool sizes in the 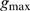-limited regime. (Note that for 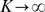, *i.e.* in the absence of feedback inhibition, there is no steady-state solution of Eq. 3 for 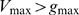 and the pool size 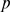 grows without limit. In reality, other processes, e.g. leakage, degradation, or constraints, e.g. thermodynamics [11], may limit steady-state intracellular metabolite-pool sizes).

Cooperative or ultrasensitive feedback (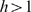) can restrict the metabolite-pool size without sacrificing growth rate. In the 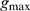-limited regime, ultrasensitive feedback leads to a sub linear increase of pool size as 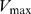 increases, 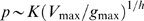. In addition, in the 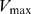-limited regime ultrasensitive feedback significantly decreases the growth-rate deficit, 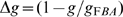, for a given value of 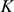, 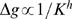. Intuitively, for a given small pool size 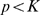, a higher Hill coefficient means weaker feedback inhibition thereby allowing more input flux and thus a higher growth rate. Consequently, for a higher Hill coefficient, a smaller inhibition constant 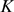 is enough to achieve a similar growth rate. Therefore, for a given growth-rate deficit 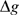, increasing the Hill coefficient 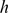 substantially reduces the metabolite-pool size in the 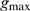-limited regime, 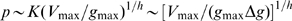, as shown for 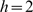 in Fig. 1B,C. Note that in Fig. 1B,C we chose feedback constants 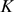 such that the resulting growth-rate is similar for the two Hill coefficients 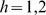.

Simple feedback regulation without ultrasensitivity has two important features: (1) simple product-feedback inhibition is enough to approach the optimal flux-balance growth rate, and (2) metabolite-pool sizes are small when growth limiting but become large when not growth limiting. These large non-growth-limiting metabolite pools can be restricted by more complex ultrasensitive feedback regulation. We test the generality of these features for various metabolic modules drawn from real metabolism.

#### Bidirectional pathway

Bidirectional pathways, such as glycolysis/gluconeogenesis, are used for switching between different nutrient sources, e.g. glucose (a 6-carbon unit) and lactate (a 3-carbon unit). At the heart of these bidirectional pathways are metabolites that are linked by two different enzymatic reactions (or pathways) of differing energetics due to different cofactor requirements, e.g. fructose-6-phosphate and fructose-1,6-bisphosphate, linked by phosphofructokinase in glycolysis and fructose-bisphosphatase in gluconeogenesis. Since these interconversions may allow cycling, limiting futile cycles between these metabolites is essential for achieving optimal growth.

Here we consider a simple module of two interconverting metabolites shown in Fig. 1D. The module has two input nutrient fluxes, 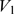 and 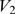, representing different sources for the same elemental nutrient (e.g. glucose and lactate for carbon), feeding into their respective intermediate metabolite pools 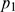 and 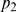. The metabolite pools can interconvert, albeit at a cost: two molecules of 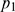 make one molecule of 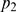 and vice versa, making futile cycling wasteful of nutrients. (For mass balance and thermodynamic consistency a low-energy waste product has to be released in each such reaction.) The interconversion fluxes between 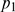 and 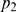 are represented by 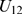 and 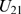, with the order of indices indicating the direction of conversion. We further assume that both metabolite pools 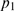 and 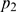 are required for growth with equal stoichiometry.

Limited availability of interconversion enzymes is modeled by the constraint on the interconversion fluxes 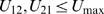 (the same constraint is used for both fluxes for simplicity). Depending on these constraints and on the maximum input fluxes, 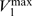 and 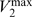, the optimal flux-balance growth rate 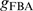 will be limited either by the input nutrient fluxes, by the interconversion fluxes, or by the maximum growth rate 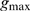. For smaller input flux into metabolite 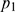, 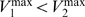, the optimal flux 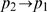 is non-zero, 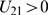, while the optimal flux 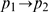 remains zero to avoid futile cycling, 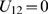. As 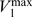 increases so that 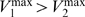, the interconversion is reversed with flux going from 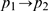. As 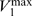 increases further, 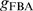 is limited either by the maximum interconversion flux 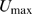 or by 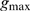. In the case when 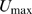 limits growth, 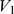 is just high enough to maximize the interconversion flux 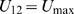. In Fig. 1E, we chose flux constraints that result in 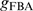 being limited by 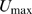 for high 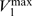 (gray lines). In all cases, the maximum growth rate is achieved by eliminating futile cycling, *i.e.* at least one of the interconversion fluxes is zero.

We compare two different regulatory schemes for this module. The simpler of the two schemes, minimal product-feedback inhibition (Min-FI) assumes feedbacks only on the input nutrient fluxes (the minimum number of feedbacks required to have a stable-steady state solution), while full product-feedback inhibition (Full-FI) assumes feedbacks on all the fluxes. Full-FI yields the following kinetic equations for the metabolite pools 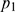 and 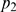,
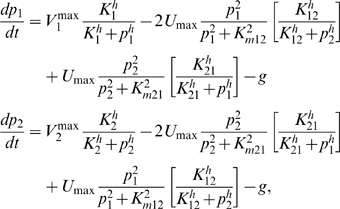
(5)where 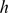 is a Hill coefficient (assumed for simplicity to be the same for all feedbacks), the 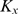, with 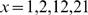, are feedback-inhibition constants, the 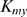, with 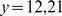, are Michaelis-Menten constants for the enzyme-substrate complexes, the exponent 2 on 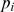 models the stoichiometry of the reactions: 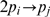, and the growth rate 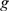 is given by Eq. 2. Note that the presence of the additional feedback terms in the Full-FI scheme (given in square brackets) makes the interconversion flux depend on a ratio of the two pool sizes, 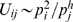, resulting in tight control of the interconversion fluxes (see below).

To achieve optimal growth, the feedback-inhibition constants are chosen according to the logic of flux-balance analysis, *i.e.* to avoid futile cycling while allowing adequate flux from non-growth-limiting metabolite pool to growth-limiting metabolite pool. To avoid futile cycling, the interconversion flux should preferentially flow from the non-growth limiting pool to the growth-limiting pool. This is achieved by choosing the Michaelis-Menten constant for each outgoing interconversion flux to be much larger than the growth-saturating substrate pool size, e.g. 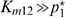. Availability of adequate input flux is accomplished by choosing the feedback constant, 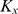, from each pool on its input flux to be much larger than the Michaelis-Menten constant, 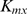 for that pool's outgoing interconversion flux, e.g. 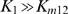.

Numerical solutions for the steady-state growth rate and metabolite-pool sizes for the two alternative regulatory schemes are shown in Fig. 1E,F. For simplicity, we have chosen parameters to make the network symmetric with respect to the two metabolites. The growth-rate deficit and the metabolite pools for the Min-FI scheme follow the same trends seen in the linear pathway: the growth-rate deficit 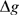 decreases as the magnitudes of feedback constants increase, the two metabolite-pool sizes switch between being small (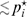) when growth-limiting and large (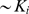) when non-growth-limiting, and the size of non-growth-limiting pool is significantly restricted by high (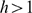) Hill coefficients. Furthermore, we find that the additional feedbacks in the Full-FI scheme better restrict the pool sizes than the Min-FI scheme, for 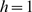.

#### Metabolic cycle

Organisms metabolize some nutrients using metabolic cycles, e.g. the TCA cycle in carbon metabolism. A metabolic cycle is a wrapped linear pathway where the end product is essential for the first step of the pathway. Consequently, the import of nutrients is slowed or stopped if there is not enough end product available. Therefore, an adequate pool of the end product must always be maintained in order to achieve optimal growth. Here we analyze a module based on the two-intermediate glutamine-glutamate nitrogen-assimilation cycle. In this cycle, ammonium (NH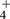) is combined with glutamate (E) to form glutamine (Q), which in turn can be combined with 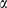-ketoglutarate to yield two molecules of glutamate.

**Figure 2:**
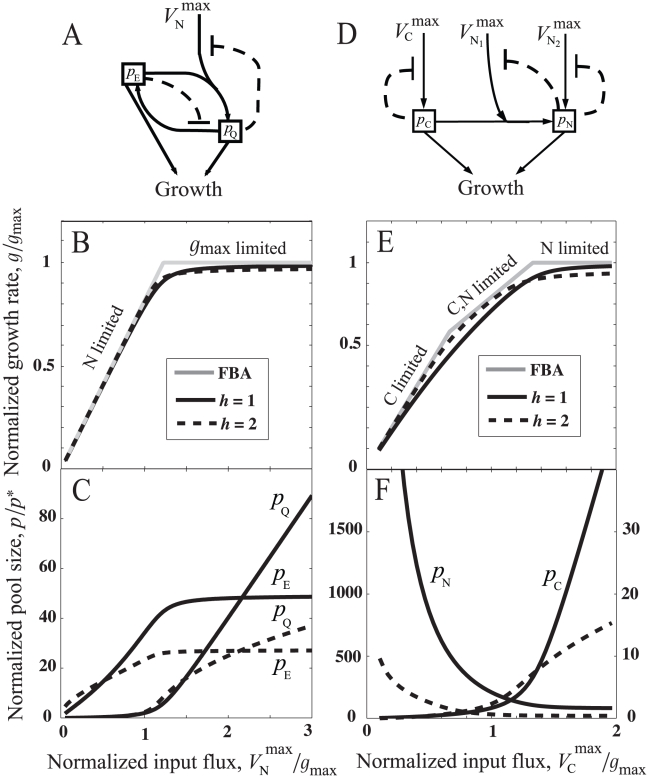
Analysis of metabolic modules: (A) metabolic cycle and (D) module for integrating carbon and nitrogen inputs. (B,C) Results for metabolic cycle module from Eq. 6: (B) 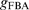, the optimal growth rate given by flux-balance analysis (FBA) (gray curve), and growth rate as a function of 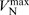 (solid and dashed curves). (C) Metabolite pool sizes 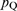 and 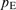 as a function of 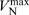. The parameters for FBA and numerical solutions: 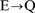 flux, 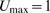, 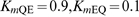, 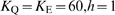 (solid curves), and 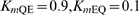, 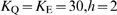 (dashed curves). In all cases, the stoichiometry factors are 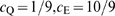 [30], consistent with the relative usage of glutamine and glutamate during growth. (E,F) Results for nutrient-integration module from Eq. 7: (E) 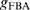 (gray curve), and growth rate as a function of 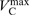 (solid and dashed curves). (F) Metabolite pool sizes 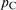 and 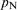 as a function of 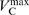. The parameters for FBA and numerical solutions: the maximum nitrogen fluxes, 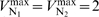, 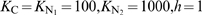 (solid curves), and 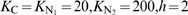 (dashed curves).

The cyclic module considered here is shown in Fig. 2A. The input nitrogen flux 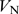 combines stoichiometrically with glutamate, with pool size 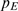, to make glutamine, with pool size 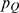. Glutamine yields two molecules of glutamate with flux 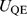, up to a maximum 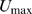, thereby completing the nitrogen assimilation cycle. We assume that both glutamine and glutamate are utilized for growth but with unequal stoichiometries, 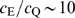 [30]. We also include the flux into glutamate from glutamine-dependent biosynthetic reactions, since these typically yield a glutamate molecule.

The optimal flux-balance growth rate 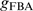 depends on the maximum input flux, 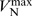, and the maximum conversion flux, 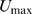, along with the maximum growth rate, 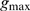. At low 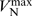, the flux-balance growth rate is proportional to the maximum input flux, 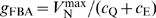 (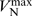-limited). As 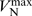 increases, the growth rate may be limited by the conversion flux, 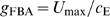 (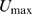-limited), or by the maximum growth rate, 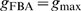 (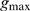-limited). In Fig. 2B we chose flux constraints that result in a 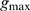-limited regime for high 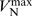 (gray lines).

As in the previous case, we compare different regulatory schemes for this module. The Min-FI schemes have only one feedback on the input flux from either glutamate or glutamine. In the Full-FI scheme, there is product-feedback inhibition of both the input flux and the conversion flux of glutamine (Q) to glutamate (E). The kinetic equations for the the Full-FI scheme are
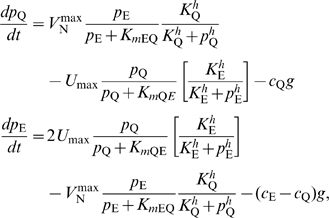
(6)where 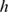 is a Hill coefficient (assumed for simplicity to be the same for all feedbacks), the 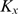, with 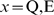 are feedback-inhibition constants, the 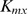, with 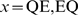, are Michaelis-Menten constants for the enzyme-substrate complexes (the order of indices indicates the direction of conversion), and the growth rate 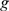 is given by Eq. 2. The kinetic equations for the Min-FI scheme with feedback on the input flux from glutamine can be recovered by dropping the terms in square brackets in Eqs. 6, and the kinetic equations for the Min-FI scheme with feedback from glutamate can then be obtained by substituting 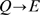 in the feedback on the input flux.

Interestingly, we find that neither of the two Min-FI schemes yield steady-state solutions that are stable in all of the three regimes: 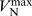-, 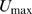-, and non-nutrient limited. In particular, stability in one regime can be guaranteed by a particular choice of Michaelis-Menten and feedback constants, but the same parameters lead to instability (one pool growing without bound or shrinking to zero) in one of the other regimes. We conclude that the metabolic cycle requires two feedbacks to assure stability, even though there is only one primary nutrient input.

For the two-feedback Full-FI scheme, to maximize the growth rate in the 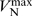-limited regime, the glutamate pool should always be saturating for the nitrogen-assimilation reaction (E
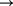
Q) so that nitrogen import is maximized. This is achieved by choosing a small Michaelis-Menten constant, 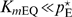. For the 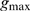-limited regime, achieving optimal growth only requires 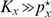. Interestingly, the Michaelis-Menten constant for the glutamine to glutamate reaction 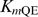 controls the relative levels of glutamate and glutamine. In Fig. 2B,C, we chose 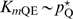 to yield results consistent with nitrogen-upshift experiments (see Text S1 and [25]).

The numerical solution of the kinetic equations for the Full-FI scheme (Fig. 2A) shows that the growth-rate deficit 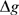 decreases as the magnitudes of feedback-inhibition constants increase and the metabolite pools are significantly reduced by high Hill coefficients. However, even though there is only one primary nutrient input like the linear pathway, the metabolic cycle requires two feedbacks to assure a stable steady state.

#### Integrating carbon and nitrogen inputs: partitioning of carbon into biomass and energy

Microorganisms integrate various nutrients to produce biomass. Since carbon sources (e.g. glucose, glycerol) are used for both biomass and energy, optimal partitioning of the carbon flux is essential for optimal growth. Here, we consider a simple module that integerates carbon and nitrogen fluxes. In *E. coli*, nitrogen in the form of ammonium (NH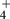) is assimilated into biomass via two pathways [31]. In the reaction catalyzed by glutamate dehydrogenase (GDH), NH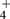 is assimilated directly into glutamate. Alternatively, in an energy-rich environment, glutamine synthetase/glutamate synthase (GS/GOGAT) form an assimilatory cycle, with NH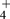 first assimilated into glutamine. This ATP-energy-dependent cycle is essential for nitrogen-limited growth of cells [31].

The metabolic module shown in Fig. 2D integrates two elemental nutrients, carbon (C) and nitrogen (N). The module has one input carbon flux 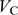 and two input nitrogen fluxes 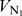 and 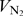 feeding into their respective intermediate metabolite pools with sizes 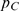 and 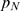. The input pathways are coupled by the carbon-dependent nitrogen flux, 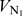, representing the GS/GOGAT cycle, which requires ATP (produced by catabolism of carbon) to import nitrogen (Fig. 2D). The other nitrogen flux, 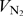, representing the ATP-independent GDH pathway, is modeled as being uncoupled from carbon metabolism (note that, in reality, both nitrogen import fluxes require also the carbon skeleton 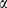-ketoglutarate). We further assume that both 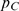 (carbon metabolites) and 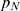 (nitrogen metabolites) are required for growth and are utilized with equal stoichiometry. Thus, proper partitioning of the carbon flux between biomass and energy for importing nitrogen is essential for achieving optimal flux-balance growth rate.

Depending on the constraints on the input fluxes: 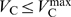, 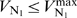, 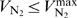, and the maximum growth rate 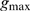, the optimal flux-balance growth 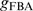 will be limited by either or both input nutrient fluxes or by 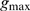 (gray curve in Fig. 2E). For small values of the maximum carbon flux, carbon will be limiting. In this regime, the carbon-dependent nitrogen flux remains zero, 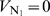, and the carbon-independent nitrogen flux stoichiometrically matches the input carbon flux, 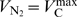. As the maximum carbon flux increases, growth becomes limited by both nitrogen and carbon – some of the carbon flux is partitioned to energy to augment the nitrogen flux. In this regime, the carbon-dependent nitrogen flux is greater than zero 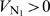, while the carbon-independent nitrogen flux is at its maximum 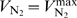. As the maximum carbon flux increases further, the growth is either limited by nitrogen availability or by 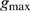 (see Text S1). In Fig. 2E we chose flux constraints that result in 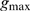-limited growth for high maximum carbon flux 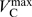.

Like previous modules, we assume product-feedback inhibition of all the input fluxes (Fig. 2D). This yields the following kinetic equations for the metabolite-pool sizes 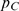 and 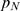,
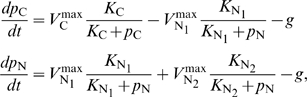
(7)where 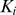, with 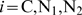, are feedback-inhibition constants, and the growth rate 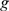 is given by Eq. 2. The carbon-dependent nitrogen flux is assumed to be unconstrained by the pool size of carbon metabolites 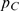, *i.e.* the affinity of the reaction for its energy substrate (ATP) is assumed to be high. The auto-regulatory negative feedbacks in the regulation scheme ensure a stable steady state.

To achieve optimal growth, the feedback-inhibition constants are chosen according to the logic of flux-balance analysis, *i.e.* the carbon-dependent nitrogen flux is turned on only after the carbon-independent nitrogen flux reaches its maximum. This is accomplished by choosing 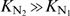.

The kinetic equations (7) are readily solved numerically for the steady-state growth rate and metabolite-pool sizes (Fig. 2E,F). As for the linear pathway, the growth-rate deficit 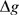 decreases as the magnitudes of the feedback constants increase. The growth-limiting metabolite pool remains small (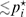) while the non-growth-limiting pool becomes large (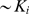) and continues to grow as its input flux increases. A metabolite pool can switch from being growth-limiting to non-growth-limiting with changes in the available input fluxes 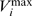. For example, in the carbon-limited regime, 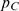 is of the order 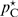 while 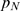 is of the order 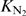 or larger (Fig. 2F). In contrast, in the nitrogen-limited regime this behavior is reversed with, 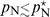 and 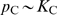 or larger. Since the carbon-derived product ATP can be used to import nitrogen, there is also a regime where both carbon and nitrogen metabolite pools are growth limiting and thus small, 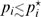. On the other hand, in the 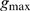-limited regime neither the carbon nor the nitrogen metabolite pool is growth limiting, consequently both pools are large (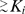) and continue to grow as their maximum input fluxes increase.

In experiments, it has been shown that the ATP-independent GDH pathway is preferred under glucose-limited growth [32], [33], which is also consistent with the optimal FBA behavior that we find in our nutrient-integration module. Furthermore, when both carbon and nitrogen are available in excess, the ATP-independent GDH pathway is largely inactive, corresponding to 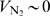 [34]. Consistent with this observation, in the 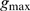-limited regime of the model, a reduction of 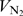 still allows for optimal growth.

The results show that simple product-feedback inhibition is sufficient to achieve the optimal flux-balance growth rate in all regimes. As for the other modules considered, larger feedback-inhibition constants improve growth rate but result in large pools of non-growth-limiting metabolites. Increasing the Hill coefficients of the feedbacks restricts pool sizes and simultaneously reduces the growth-rate deficits.

### Nitrogen assimilation in *E. coli*

Regulation of nitrogen assimilation in *E. coli* has been studied in great detail, perhaps more carefully than any other metabolic sub-network [25], [35], [36] (see also cites in [25]). As nitrogen assimilation involves both a metabolic cycle and nutrient integration, it offers a chance to examine the extent to which actual metabolic networks, beyond the much studied linear or branched biosynthetic pathways, are regulated by feedback inhibition circuits of the sort that we hypothesize above.

Our mathematical analysis of metabolic cycle and nutrient integration suggest a simple regulation scheme that allows near optimal steady-state growth. For the nitrogen assimilation GS/GOGAT cycle the analysis suggests feedback inhibition by glutamine and glutamate on GS and GOGAT, respectively. Feedback inhibition of GS by glutamine is well known. It does not involve standard allostery, but instead a bicyclic cascade of covalent modifications [37]. Interestingly, consistent with our suggestion that ultrasensitive feedback might be necessary for adequate control of metabolite pool sizes, it has been proposed that the purpose of this bicyclic cascade is to yield ultra-sensitive feedback [38]. Feedback inhibition of GOGAT by glutamate, in contrast, had not been explicitly considered until recent efforts at data-driven modeling of the network [25]. These efforts revealed that such feedback inhibition is essential to obtain models that match experimental data. Furthermore, examination of older literature reveals biochemical evidence for such feedback inhibition: glutamate and aspartate both inhibit GOGAT activity [39]. The effect of glutamate is an example of standard product inhibition of an enzyme, and was considered initially insignificant due to the high inhibition constant (*i.e.*, the feedback is weak). However, given the large cellular pool size of glutamate (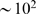 mM), the high inhibition constant is appropriate (indeed matching our expectation that large inhibition constant values are required to obtain near-optimal growth, with the associated consequence of large metabolite pool sizes). Aspartate is a direct product of glutamate, and provides further feedback essentially as a glutamate surrogate.

For the ATP-independent nitrogen flux via GDH the analysis suggests feedback inhibition of GDH by the key nitrogen intermediates, glutamine and glutamate, which is again consistent with biochemical studies of purified GDH enzyme and with the existence of product inhibition of all enzymatic reactions [40], [41].

A prediction from our analysis is that large changes in metabolite pools will occur upon the onset of nutrient limitation. This also agrees well with experimental observations. For example, consider the dynamics of 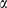-ketoglutarate and glutamine, the carbon skeleton and the most nitrogen-rich product of central nitrogen metabolism. 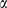-ketoglutarate is part of the TCA cycle, and many TCA cycle metabolites show similar patterns to its temporal response during nitrogen limitation and re-addition [25]. Accordingly, we consider the 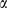-ketoglutarate level as an indicator of available carbon (specifically, carbon in the TCA cycle). Glutamine levels have been shown to correlate well with growth rate under nitrogen limitation [36], and accordingly we consider glutamine levels to indicate available nitrogen.

**Figure 3:**
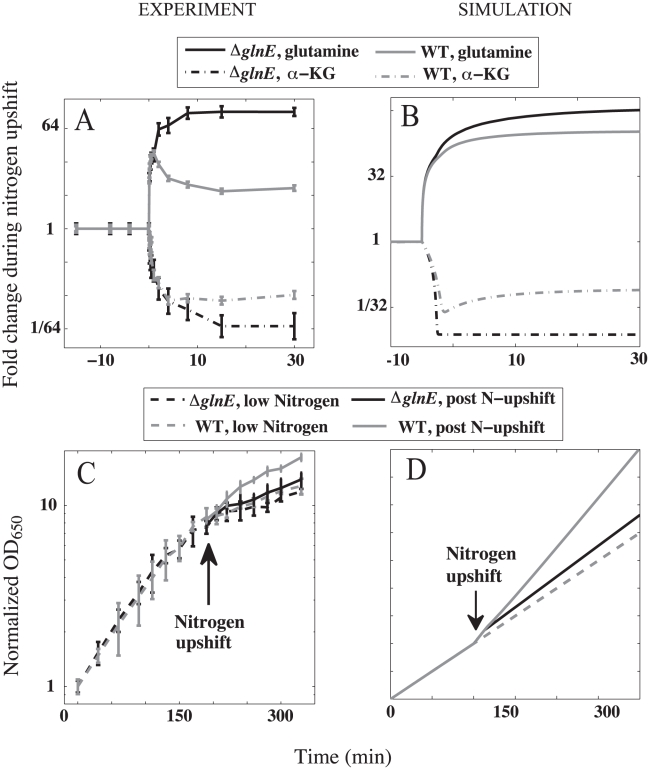
Nutrient-switching experiment with feedback-knockout 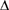 *glnE* (FG 1114) and WT strains of *E. coli* and simulations of analogous modules. (A) Fold changes in key carbon and nitrogen intermediates, 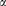-ketoglutarate (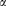-KG) and glutamine, under nitrogen upshift. (B) Simulated fold changes in the carbon and nitrogen intermediates 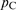 and 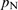 in the two strains after nitrogen upshift, which is applied by changing both maximum nitrogen input fluxes 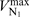 and 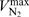 from 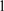 to 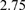; the maximum carbon input flux is fixed at 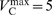. (C) Normalized growth curves. Measured optical density (OD) are normalized by OD at 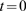 for each experiment. (D) Simulated growth curve for modules analogous to the two strains. All data presented are averages and standard error of multiple (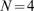 for wild-type, 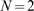 for 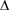 *glnE*) independent experiments conducted on separate days.

Fig. 3A shows the experimental metabolite pool size dynamics following nitrogen limitation and subsequent upshift for wild-type *E. coli*, as well as *E. coli* lacking the covalent modification enzyme responsible for feedback inhibition of glutamine synthetase (GS) by glutamine (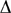 *glnE*). The steady-state metabolite pool sizes of the two strains are nearly identical before the nitrogen upshift; however, upon nitrogen upshift, the fold changes in both 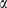-ketoglutarate and glutamine are amplified in the feedback-defective strain compared to the WT strain. Moreover, after the nitrogen upshift, large amounts of extracellular amino acids, including glutamine and glutamate, were measured in cultures of the feedback-defective strain consistent with unregulated nitrogen assimilation (Fig. 3 in Text S1). These observations are consistent with simulations based on our simple feedback model (Fig. 3B). Furthermore, we find metabolite pool dynamics observed under nitrogen-limited growth to also be consistent with our model [42] (see Text S1).

Within our model, the WT strain is described by the module with all three feedbacks present (Fig. 2D), while the feedback-defective strain is described by the same basic module but without the feedback on carbon-dependent nitrogen input flux 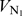. As a simulation of the experiment, we started the two modules at steady state in the nitrogen-limited regime, and suddenly increased nitrogen availability by simultaneously increasing the two nitrogen maximum input fluxes 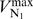 and 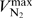, thereby shifting the modules to the non-nutrient limited (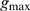-limited) regime. To achieve steady state in our model for the feedback-defectve strain, we assumed a leakage flux for the large nitrogen intermediate pool 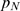 (see Text S1 for equations with leakage).

Some of the system's dynamics, in particular the overshoot of glutamine in the wild-type strain, are not captured by our simple feedback model. Generally, time-delay in the feedback may result in an overshoot in a feedback-inhibited system. This is consistent with the specific implementation of feedback by glutamine on GS: a cascade of covalent modification reactions which occur on the 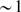 min timescale, with the overshoot occurring in the period where nitrogen assimilation outraces the feedback mechanism.

We also compared the growth rate response of the wild-type and feedback-defective strains to relief of nitrogen limitation. Consistent with experimental results, the simulations predicted a bigger increase in the growth rate in the WT strain than in the feedback-defective strain following nitrogen upshift (Fig. 3C,D). In the simulation, the reason for the slower growth in the feedback-defective strain post nitrogen up-shift is excessive drainage of the carbon metabolite pool (e.g., 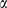-ketoglutarate) by unregulated nitrogen uptake in the feedback-defective strain. Whether such drainage of a valuable carbon species is the real reason in live cells is not clear, however. An alternative possibility is that the excessive accumulation of glutamine causes osmotic imbalance. This alterative, while not quantitatively included in our model, is nevertheless consistent with the role of feedback inhibition as a homeostatic regulatory mechanism.

## Discussion

Understanding metabolism and its regulation have long been central goals of biochemistry. Recently, flux-balance analysis (FBA), a constraint-based computational approach, has been used to predict the optimal metabolic fluxes and growth rates of microorganisms in different environments. In several cases, in particular involving *E. coli*, the FBA-predicted optima agree remarkably well with experiments [1], [34], raising the question “for cells to realize optimal growth how complex must metabolic regulation be?” We have addressed this question using a set of representative metabolic modules. We find that, in all the cases studied, simple product-feedback inhibition is enough to achieve nearly optimal growth. Furthermore, the divergence from optimality becomes arbitrarily small as the feedback-inhibition constants are increased.

An important trade-off is that larger inhibition constants result in larger pool sizes of non-growth-limiting metabolites, which can be detrimental to growth. However, ultrasensitive feedback mechanisms (*i.e.* those with high Hill coefficients) can substantially restrict these pool sizes; the higher the Hill coefficient of the feedbacks, the smaller the increase in pool size required to achieve the same degree of inhibition. This suggests that the need for ultrasensitive mechanisms to control metabolite pool sizes may account for some of the complexity found in metabolic regulation in real cells at both the transcriptional and post-transcriptional levels.

Can we hope to gain insight into real metabolism using the very simple models we studied? To address this question we examined the nitrogen assimilation network in *E. coli*, which involves both nutrient integration and a metabolic cycle. First, we found the feedback regulation scheme proposed by our mathematical analysis of representative modules aligns closely with the known regulation of the network. Second, we found reasonable agreement between simulations based on our simple feedback models and actual experimental results, for both wild type and feedback-defective *E. coli*. Comparing strains with different regulatory schemes allowed us to directly ask the question “is product-feedback inhibition essential for achieving optimal growth?” At least in the case of nitrogen up-shift, both our simulations and experimental data argue that it is: the feedback-defective strain grew substantially slower than wild type after the up-shift.

One of the central predictions of our feedback framework is that pool sizes will be large for non-growth-limiting metabolites. Since few metabolites are growth-limiting under any nutrient condition, the cells are likely to have large pools of multiple metabolites under a wide range of conditions. Therefore, we need to consider the possible impact of large pool sizes on cell physiology. Can large sizes of metabolite pools be detrimental to the well-being of cells? In fact, many metabolic intermediates, such as glyoxylate and formaldehyde, are toxic at high concentrations. Even the biosynthetic end-products required for growth (e.g. amino acids, nucleotides, etc.) can be detrimental to a cell's growth at high enough concentrations. Metabolites at high concentration can interact nonspecifically with various enzymes and disrupt metabolic reactions [43]. Furthermore, metabolite pools contribute to intracellular osmolarity and consequently to the osmotic pressure inside cells. Dedicated mechanisms to respond to osmotic stress have evolved in microorganisms, reflecting the harmful effects of osmotic imbalance [44]–[46]. For *E. coli*, the growth rate is maximized in conditions corresponding to external osmotic pressures of around 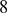 atm [45], [46]. Furthermore, the turgor pressure has been estimated to be around 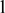 atm [47] in an AFM study of the magnetotactic Gram negative bacteria *Magnetospirillium gryphiswaldense*. Consequently, the internal osmotic pressure is thought to be around 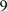 atm, which corresponds to an effective concentration of 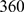 mM of solute. Recent measurements have revealed that some metabolite pools can become very large, such as fructose-1,6-bisphosphate (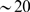 mM) and glutamate (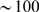 mM) [48]. These large metabolite pools could contribute significantly to the overall internal osmotic pressure of the cells. In general, pools that are large even when growth-limiting will potentially be very large when non-growth-limiting and may cause osmotic imbalance. Such pools in particular may require ultrasensitive feedback mechanisms to restrict their sizes. Experimental manipulation of feedback sensitivities (e.g. by enzyme mutation, knockout of enzymes involved in covalent modification cascades, etc.) should help shed light on the role of ultrasensitive feedback mechanisms.

**Figure 4:**
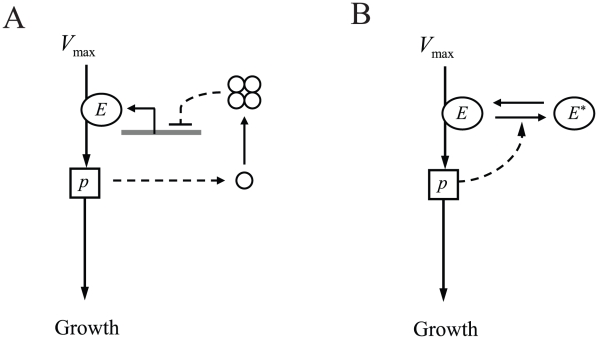
Examples of transcriptional and post-transcriptional regulation schemes for ultrasensitive feedback. (A) Cooperative transcriptional regulation. The product of enzyme 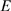, with pool size 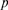, allosterically controls the activity of transcription factors (circles) that cooperatively regulate the expression of enzyme 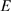. (B) Zeroth-order ultrasensitivity via post-transcriptional covalent modification. 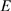 is the active enzyme while 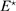 is the modified inactive enzyme. The product of enzyme 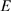 activates conversion of 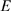 to 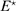 (or equivalently inhibits conversion of 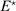 to 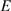) where both the reactions are zeroth-order, *i.e.*, saturated with respect to 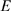 and 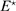 concentrations. Dashed lines represent regulatory connections, where the bar is used to represent inhibition.

Ultrasensitivity is a common feature of feedback inhibition. At the transcriptional level, multiple promoter binding sites along with other cooperative mechanisms like DNA looping yield ultrasensitive responses [49] (Fig. 4A). The response time for transcriptional feedback is limited by protein degradation (and dilution), which in microorganisms is typically of the order of tens of minutes to hours. Metabolite-pool sizes, on the other hand, may change in just few seconds, e.g. the glutamine pool increased by over 10-fold in 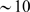 seconds in the nutrient-switching experiment described above. The fast dynamics of metabolite-pool sizes suggests the need for fast feedback mechanisms. Fast regulation can be realized through various post-translational mechanisms – allosteric regulation of protein aggregates, e.g. ATP molecules bind cooperatively to a homodimer of pantothenate kinase [50], competition, e.g. Wee1 regulation of Cdk1 [51], or covalent modifications, e.g. reversible phosphorylation of isocitrate dehydrogenase [52] and the bicyclic cascade of covalent modifications in glutamine regulation [26] (Fig. 4B). Thus, the need for fast ultrasensitive feedback mechanisms may be a key driver of the observed complexity in metabolic regulation.

Our study of simple representative metabolic modules is an attempt to identify the design principles underlying the regulatory mechanisms that optimize metabolic function, such as biomass production [53]. In addition to highlighting general lessons in metabolic regulation, our analysis raises new fundamental questions. How many feedbacks are required in a metabolic network, in particular the metabolic network of a real cell? What principles, in addition to optimal growth and stability, guide the evolutionary selection of feedbacks and feedback mechanisms? Has the complexity and dynamics of the cellular environment led to additional constraints on feedback strategies? And finally, given the apparent sufficiency of feedback inhibition, why are other regulatory motifs, such as allosteric enzyme activation, also found in metabolism? Further experiments in which metabolic feedbacks are eliminated, modified, and/or rewired, in concert with additional theoretical analyses, should facilitate answering these questions.

## Materials and Methods

The analyses were carried out using kinetic equations (Eqs. 3, 5, 6, 7). The equations account for the concentration of each component in the metabolic modules and the steady-state solutions were numerically obtained using MATLAB. For details on the flux-balance analysis (FBA) see Text S1.

## Supporting Information

Text S1 Additional information.
(0.25 MB PDF)
