# Supplementary material for: Achieving Optimal Growth through Product Feedback Inhibition in Metabolism
Source: PLoS Comput Biol. 2010 Jun 3;6(6):e1000802. doi: 10.1371/journal.pcbi.1000802 (PMC2880561; doi:10.1371/journal.pcbi.1000802)
Supplement: Text S1 — Additional information. (0.25 MB PDF) [file pcbi.1000802.s001.pdf]

Text S1 - Additional Information

Sidhartha Goyal[a], Jie Yuan[b], Thomas Chen[c], Josh D. Rabinowitz[b], and Ned S. Wingreen[d]
[a]*Department of Physics,* [b]*Department of Applied Mathematics,* [c]*Department of Chemistry, and* [d]*Department of Molecular Biology, Princeton University, Princeton, NJ 08544*

I. MODEL

We present here flux-balance analysis (FBA) for the four metabolic modules considered in the main text (MT). We also analytically calculate asymptotic expressions for growth rate and pool size for the linear-chain module including feedback. Furthermore, complete analysis of the feedback knock-out strain is also presented here.

A. Linear Chain

The flux-balance condition at steady state for the linear-chain module shown in Fig. 1 of the main text is given by

$$g = V. \tag{1}$$

This along with the linear constraints on input flux and growth rate,

$$\begin{aligned} V &\leq V_{\max} \\ g &\leq g_{\max}, \end{aligned} \tag{2}$$

yield the following for the FBA growth rate,

$$g_{\text{FBA}} = \begin{cases} V_{\max}, & V_{\max} \leq g_{\max} \\ g_{\max}, & V_{\max} \geq g_{\max} \end{cases} \tag{3}$$

where g_{FBA} is the maximum allowed growth rate consistent with all the constraints.

Including feedback regulation, the kinetic equation for the metabolite-pool size p is

$$\frac{dp}{dt} = V_{\max}\frac{K^h}{K^h + p^h} - g_{\max}\frac{p}{p + p^\star}, \tag{4}$$

where p is the metabolite-pool size, h is a Hill coefficient and K is an inhibition constant. The steady-state pool size therefore satisfies

$$V_{\max}\frac{K^h}{K^h + p^h} = g_{\max}\frac{p}{p + p^\star}$$

$$\Longrightarrow (p/K)^{h+1} + (p/K)(1 - \tilde{V}) - \tilde{V}p^\star/K = 0 \tag{5}$$

where $\tilde{V} = V_{\max}/g_{\max}$. It is straightforward to calculate the asymptotic behavior of p in the two asymptotic FBA regimes: flux-limited ($\tilde{V} \ll 1$) and growth-limited ($\tilde{V} \gg 1$). We assume a large feedback-inhibition constant $K \gg p^\star$, this yields

$$p \approx \begin{cases} p^\star \tilde{V}, & \tilde{V} \ll 1 \\ K\tilde{V}^{1/h}, & \tilde{V} \gg 1 \end{cases} \tag{6}$$

where p is the steady-state metabolite pool; we keep terms to leading order in K.

In the $V_{\max}$-limited regime, i.e. where $\tilde{V} \ll 1$, the growth rate is

$$\begin{aligned} g &= V_{\max}\frac{K^h}{K^h + p^h} \\ &\approx V_{\max}\left(1 - \frac{p^h}{K^h}\right) \end{aligned} \tag{7}$$

where we have used $p \ll K$. This yields for the growth-rate deficit

$$\begin{aligned}\Delta g &= 1 - \frac{g}{g_{\rm FBA}} &(8)\\ &\approx \frac{p^h}{K^h} &(9)\\ &= \tilde{V}^h \frac{p^{\star h}}{K^h}. &(10)\end{aligned}$$

In the $g_{\max}$-limited regime, $\tilde{V} \gg 1$, where $p \sim K$, the growth rate is

$$\begin{aligned}g &= g_{\max} \frac{p}{p + p^\star}\\ &\approx g_{\max}\left(1 - \frac{p^\star}{p}\right), &(11)\end{aligned}$$

yielding for the growth-rate deficit

$$\begin{aligned}\Delta g &\approx \frac{p^\star}{p}\\ &= \frac{p^\star}{K\tilde{V}^{1/h}}. &(12)\end{aligned}$$

B. Bidirectional pathway

The flux-balance condition at steady state for the bidirectional module shown in Fig. 3 of the main text is given by

$$\begin{aligned}c_1 g &= V_1 - 2U_{12} + U_{21}\\ c_2 g &= V_2 - 2U_{21} + U_{12}, &(13)\end{aligned}$$

where c_1, c_2 give the stoichiometry of utilization of the two intermediates. These along with the linear constraints on input fluxes,

$$\begin{aligned}V_1 &\leq V_1^{\max}\\ V_2 &\leq V_2^{\max}\\ U_{12,21} &\leq U_{\max}, &(14)\end{aligned}$$

are used to calculate maximum growth-rate $g_{\rm FBA}$, first for $V_1^{\max}/c_1 \leq V_2^{\max}/c_2$

$$g_{\rm FBA} = \begin{cases} \frac{V_1^{\max}+U_{\max}}{c_1}, & \frac{V_1^{\max}}{c_1} \leq \left[\frac{V_2^{\max}}{c_2} - U_{\max}\left(\frac{1}{c_1}+\frac{2}{c_2}\right)\right] \\ \frac{2V_1^{\max}+V_2^{\max}}{2c_1+c_2}, & \left[\frac{V_2^{\max}}{c_2} - U_{\max}\left(\frac{1}{c_1}+\frac{2}{c_2}\right)\right] \leq \frac{V_1^{\max}}{c_1} \leq \frac{V_2^{\max}}{c_2}. \end{cases} \quad (15)$$

Due to symmetry, the FBA growth rate for $V_1^{\max}/c_1 \geq V_2^{\max}/c_2$ can be found by exchanging the $1 \leftrightarrow 2$ indices. The above calculation assumes growth is limited by nutrients; if the growth is limited by $g_{\max}$, then $g_{\rm FBA} = g_{\max}$.

C. Metabolic cycle

The flux-balance condition at steady state for the metabolic cycle shown in in Fig. 4 of the main text is given by

$$\begin{aligned}c_{\rm Q} g &= V_{\rm N} - U\\ (c_{\rm E} - c_{\rm Q}) g &= 2U - V_{\rm N} &(16)\end{aligned}$$

where $c_{\rm Q}$, $c_{\rm E}$ are the stoichiometry of utilization of glutamine and glutamate, respectively. The growth rate is maximized within the following linear constraints on fluxes,

$$\begin{aligned}V_{\rm N} &\leq V_{\rm N}^{\max}\\ U &\leq U_{\max}, &(17)\end{aligned}$$

which yields

$$g_{\rm FBA} = \begin{cases} \frac{V_{\rm N}^{\rm max}}{c_{\rm Q}+c_{\rm E}}, & \frac{V_{\rm N}^{\rm max}}{c_{\rm Q}+c_{\rm E}} \leq \frac{U_{\rm max}}{c_{\rm E}} \\ \frac{U_{\rm max}}{c_{\rm E}}, & \frac{V_{\rm N}^{\rm max}}{c_{\rm Q}+c_{\rm E}} \geq \frac{U_{\rm max}}{c_{\rm E}}. \end{cases}$$

The above calculation assumes growth is limited by nutrients; if the growth is limited by $g_{\rm max}$, then $g_{\rm FBA} = g_{\rm max}$.

D. Integration of two different nutrient inputs

The flux-balance condition at steady state for the carbon-nitrogen module shown in Fig. 1 of the main text is given by

$$\begin{aligned} c_{\rm C} g &= V_{\rm C} - V_{\rm N_1}, \\ c_{\rm N} g &= V_{\rm N_1} + V_{\rm N_2}, \end{aligned} \tag{18}$$

where we have included facors $c_{\rm C}, c_{\rm N}$ giving the stoichiometry of utilization of the two intermediates. Assuming the following linear constraints on input fluxes,

$$\begin{aligned} V_{\rm C} &\leq V_{\rm C}^{\rm max} \\ V_{\rm N_1} &\leq V_{\rm N_1}^{\rm max} \\ V_{\rm N_2} &\leq V_{\rm N_2}^{\rm max}, \end{aligned} \tag{19}$$

yields the FBA optimal growth rate

$$g_{\rm FBA} = \begin{cases} \frac{V_{\rm C}^{\rm max}}{c_{\rm C}}, & \frac{V_{\rm C}^{\rm max}}{c_{\rm C}} \leq \frac{V_{\rm N_2}^{\rm max}}{c_{\rm N}} \\ \frac{V_{\rm C}^{\rm max}+V_{\rm N_2}^{\rm max}}{c_{\rm C}+c_{\rm N}}, & \frac{V_{\rm N_2}^{\rm max}}{c_{\rm N}} \leq \frac{V_{\rm C}^{\rm max}}{c_{\rm C}} \leq \left(\frac{1}{c_{\rm C}} + \frac{1}{c_{\rm N}}\right) V_{\rm N_1}^{\rm max} + \frac{V_{\rm N_2}^{\rm max}}{c_{\rm N}} \\ \frac{V_{\rm N_1}^{\rm max}+V_{\rm N_2}^{\rm max}}{c_{\rm N}}, & \frac{V_{\rm C}^{\rm max}}{c_{\rm C}} \geq \left(\frac{1}{c_{\rm C}} + \frac{1}{c_{\rm N}}\right) V_{\rm N_1}^{\rm max} + \frac{V_{\rm N_2}^{\rm max}}{c_{\rm N}} \end{cases} \tag{20}$$

The above expressions apply if growth in limited by nutrients, otherwise the growth rate is limited by $g_{\rm max}$, i.e. $g_{\rm FBA} = g_{\rm max}$.

Model for feedback knock-out strain. In the feedback knock-out (KO) strain, the negative feedback from glutamine on its own biosynthetic enzyme glutamine synthetase is absent. The module describing the KO strain is the basic carbon and nitrogen combining module without the feedback on the carbon-dependent nitrogen input flux $V_{\rm N_1}$. Furthermore, we allow leakage out of the cell from the nitrogen intermediate $p_{\rm N}$. This yields the following kinetic equations for metabolite pools $p_{\rm C}$ and $p_{\rm N}$,

$$\begin{aligned} \frac{dp_{\rm C}}{dt} &= V_{\rm C}^{\rm max} \frac{K_{\rm C}}{K_{\rm C} + p_{\rm C}} - V_{\rm N_1}^{\rm max} - g \\ \frac{dp_{\rm N}}{dt} &= V_{\rm N_1}^{\rm max} + V_{\rm N_2}^{\rm max} \frac{K_{\rm N_2}}{K_{\rm N_2} + p_{\rm N}} - V_{\rm L} \frac{p_{\rm N}}{K_{\rm L} + p_{\rm N}} - g, \end{aligned} \tag{21}$$

where h is a Hill coefficient (assumed for simplicity to be the same for all feedbacks), the K_i, with $i = {\rm C}, {\rm N_1}, {\rm N_2}$, are feedback-inhibition constants, and the growth rate g is given by Eq. 2 in MT. The auto-regulatory negative feedback on carbon flux and the leakage of $p_{\rm N}$ ensure a steady state, which is guaranteed to be stable by the 1:1 stoichiometry (Goyal and Wingreen, 2007).

II. EXPERIMENTS

A. Nitrogen starvation

One prediction from our analysis is that large changes in metabolite pools will occur upon the onset of nutrient limitation. Such large changes in metabolite-pool sizes have recently been observed in nutrient switching experiments

with *Escherichia coli* (Brauer *et al*, 2006). When cells growing on filters were moved from a minimal media (no nutrient limitation) agar plate to a no NH_4^+ (nitrogen limited) agar plate, the glutamine pool (a nitrogen intermediate) decreased by almost 64 fold, while the pool of α-ketoglutarate (a carbon intermediate) increased by almost 16 fold (Fig. 1). Furthermore, the changes in metabolite pools were monotonic over short times ($\lesssim$ 1 hour). However, pools of other metabolites such as ATP and glutamate, which are known to fulfill other functions in cell, did not change much after the nutrient switch (ATP serves as the energy currency of the cell and glutamate is the dominant counter-ion for potassium).

Despite the complexity of the real metabolic network, we find that our simple module for combination of carbon and nitrogen predicts pool size dynamics qualitatively similar to the measurements (see Fig. 1 inset). To make the analogy between our simple module and the real metabolic network, we identify our carbon intermediate $p_{\rm C}$ with α-ketoglutarate and our nitrogen intermediate $p_{\rm N}$ with glutamine. As a simulation of the experiment, we start the carbon-nitrogen module in steady state in the non-nutrient limited regime, and at time $t = 0$, reduce the input nitrogen availability by simultaneously reducing tenfold the two maximum input nitrogen fluxes $V_{\rm N_1}^{\rm max}$ and $V_{\rm N_2}^{\rm max}$. The metabolite pools then evolve dynamically as per Eq. 7 in MT. Consistent with the experimental data, the carbon intermediate pool $p_{\rm C}$ increases monotonically while the nitrogen intermediate pool $p_{\rm N}$ decreases monotonically before approaching new steady states. Note that the consistency between simulations and the experimental data degrades at long times ($\gtrsim$ 1 hour). In order to understand such long-time behavior of the metabolite pools one would have to include the effects of genetic regulation on the various enzyme concentrations.

B. Nitrogen up-shift

Interestingly, the leakage of key nitrogen intermediates, glutamine and glutamate, in the feedback-defective strain following nitrogen upshift depends on the nitrogen source used in the media. Particularly, both glutamate and glutamine leak for ammonia as nitrogen source while only glutamine leaks out for proline (also see Kustu *et al.*, 1984) as nitrogen source (Fig. 3). In cells growing in proline media, synthesis of glutamate from glutamine is suppressed as proline directly substitutes for glutamate. Consequently, the metabolic cycle between glutamine and glutamate is broken in the proline media, *i.e.* the reaction from glutamate to glutamine is active and the reaction from glutamine to glutamate is inactive. Therefore, in the feedback-defective strain, following nitrogen upshift in proline media, additional glutamate (coming from proline) is readily converted into glutamine leading to large production of glutamine. However, in ammonia media, the full metabolic cycle is active and therefore both glutamine and glutamate are synthesized at high rates following the nitrogen upshift.

---

[1] Bajad SU, Lu W, Kimball EH, Yuan J, Peterson C, et al. (2006) Separation and quantitation of water soluble cellular metabolites by hydrophilic interaction chromatography-tandem mass spectrometry. *J Chromatogr A* **1125:** 76-88.

[2] Bennett BD, Kimball EH, Gao M, Osterhout R, Van Dien SJ, Rabinowitz JD (2009) Nature Chemical Biology Absolute metabolite concentrations and implied enzyme active site occupancy in Escherichia coli. *Nat Chem Biol.* **8:** 593-599

[3] Brauer MJ, Yuan J, Bennett BD, Lu W, Kimball EH, Botstein D, and Rabinowitz JD (2006) Conservation of the metabolomic response to starvation across two divergent microbes. *PNAS* **103:** 19302-07

[4] Goyal S and Wingreen NS (2007) Growth-induced instability in metabolic networks. *Phy Rev Lett* **98:**138105

[5] Gutnick D, Calvo JM, Klopotowski T, Ames BN (1969) Compounds which serve as the sole source of carbon or nitrogen for Salmonella typhimurium LT-2. *J Bacteriol* **100:** 215-219

[6] Kraml CM, Zhou D, Byrne N, McConnell O (2005) Enhanced chromatographic resolution of amine enantiomers as carbobenzyloxy derivatives in high-performance liquid chromatography and supercritical fluid chromatography. *J Chromatogr A* **1100:** 108-115

[7] Kustu S, Hirsehman J, Burton D, Jelesko J, and Meeks JC (1984) Covalent modification of bacterial glutamine synthetase: physiological significance *Mol Gen Genet* **197:**309-317

[8] Ikeda TP, Shauger AE, and Kustu S (1996) Salmonella typhimurium apparently perceives external nitrogen limitation as internal glutamine limitation. *J Mol Biol.* **259(4):** 589-607

[9] Lu W, Bennett BD, Rabinowitz JD (2008) Analytical strategies for LC-MS-based targeted metabolomics. *J Chromatogr B Analyt Technol Biomed Life Sci*

[10] Nogle LM, Mann CW, Watts WL, Jr., Zhang Y (2006) Preparative separation and identification of derivatized beta-methylphenylalanine enantiomers by chiral SFC, HPLC and NMR for development of new peptide ligand mimetics in drug discovery. *J Pharm Biomed Anal* **40:** 901-909

[11] Soupene E, van Heeswijk WC, Plumbridge J, Stewart V, Bertenthal D, et al. (2003) Physiological studies of Escherichia coli strain MG1655: growth defects and apparent cross-regulation of gene expression. *J Bacteriol* **185:** 5611-5626

[12] Wohlhueter RM, Schutt H, and Holzer H (1973) Regulation of glutamine synthetasein vivo in *E. coli.* In *The Enzymes of Glutamine Metabolism*, Prusiner S and Stadtman ER. (eds) pp 45-64. Academic Press, New York, USA
[13] Yan D, Ikeda TP, Shauger AE, and Kustu S (1996) Glutamate is required to maintain the steady-state potassium pool in Salmonella typhimurium. *PNAS* **93(13):** 6527- 6531
[14] Yuan J, Bennett B, Rabinowitz J (2008) Kinetic flux profiling for quantitation of cellular metabolic fluxes. *Nature Protocols* **3:** 1328 - 1340

III. FIGURES

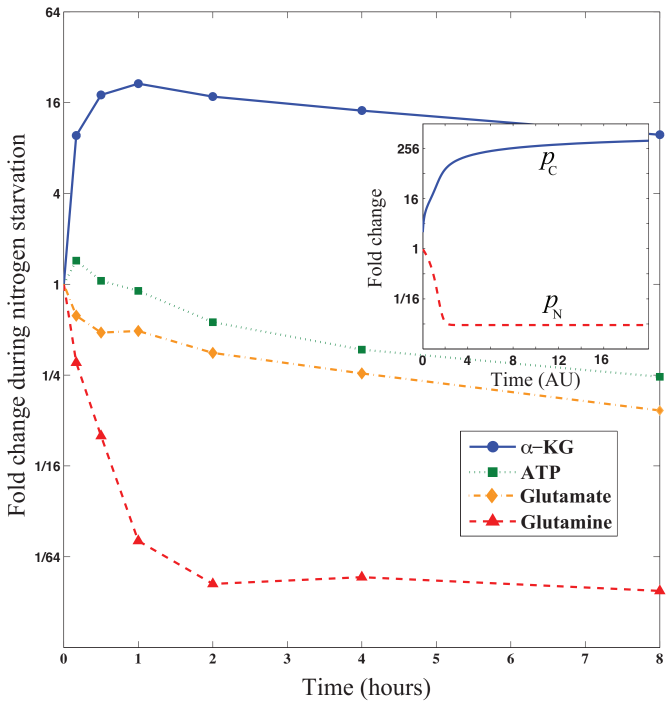

FIG. 1: **Changes in key metabolic pool concentrations in *E. coli* under nitrogen starvation. Upon nitrogen starvation, α-ketoglutarate (α-KG) (a carbon intermediate) increases monotonically, while free glutamine (a nitrogen intermediate) decreases monotonically, while ATP (derived from carbon metabolism) and free glutamate (a nitrogen intermediate) do not change more than twofold for times $\lesssim 1$ hour (Brauer *et al*, 2006). Inset: Fold changes in the carbon and nitrogen intermediates $p_{\rm C}$ and $p_{\rm N}$ after the maximum input nitrogen fluxes $V_{\rm N_1}^{\rm max}$ and $V_{\rm N_2}^{\rm max}$ are reduced tenfold in our carbon-nitrogen module (Eq. 7 in Main Text). The input fluxes before nitrogen starvation are $V_{\rm C}^{\rm max} = 3$, $V_{\rm N_1}^{\rm max} = V_{\rm N_2}^{\rm max} = 1.5$, the growth-saturating concentrations are $p_{\rm C}^{\star} = p_{\rm N}^{\star} = 1$, and the feedback-inhibition constants are $K_{\rm C} = K_{\rm N_1} = 100$, $K_{\rm N_2} = 1000$.**

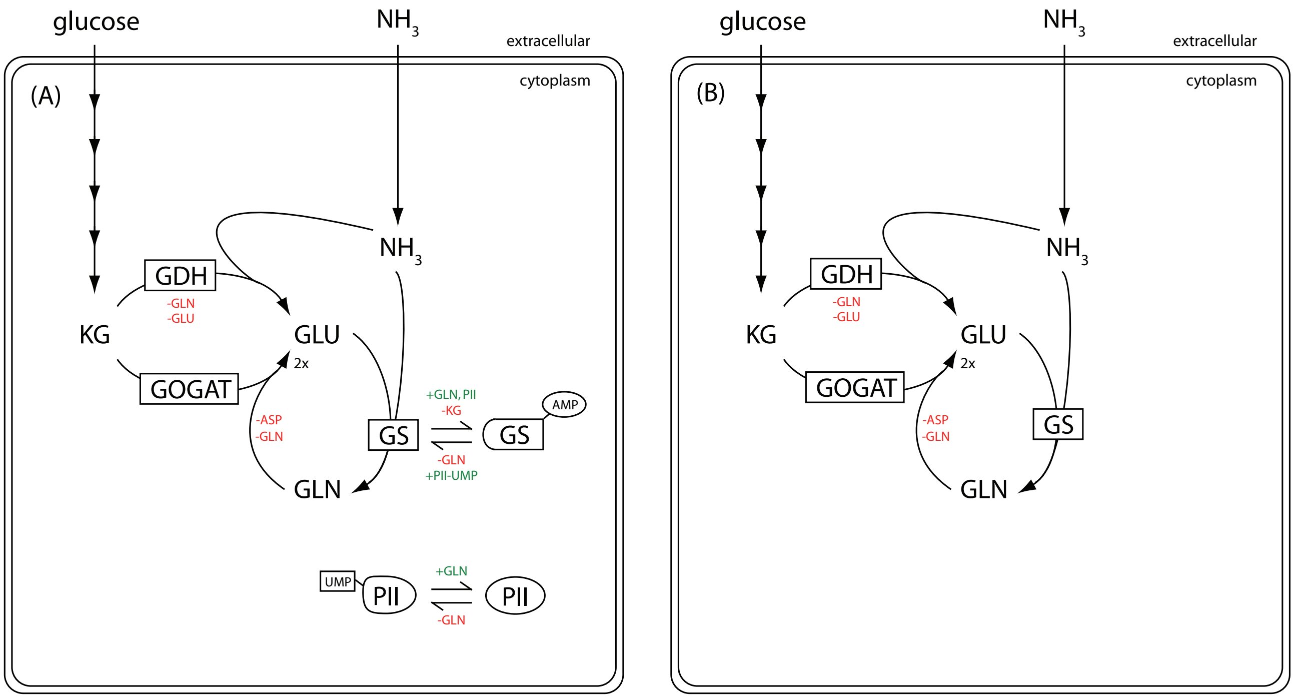

FIG. 2: **Key elements of carbon-nitrogen metabolism for (A) WT and (B) feedback-knockout $\Delta glnE$ (FG 1114) stains of** ***E. coli*****. Metabolites: KG - α-ketoglutarate, GLU - glutamate, and GLN - glutamine. Enzymes: GDH - glutamate dehydrogenase, GOGAT - glutamate synthase, and GS - glutamine synthetase. The regulation of reactions by metabolites is mentioned in red (green) representing negative (positive) regulation.**

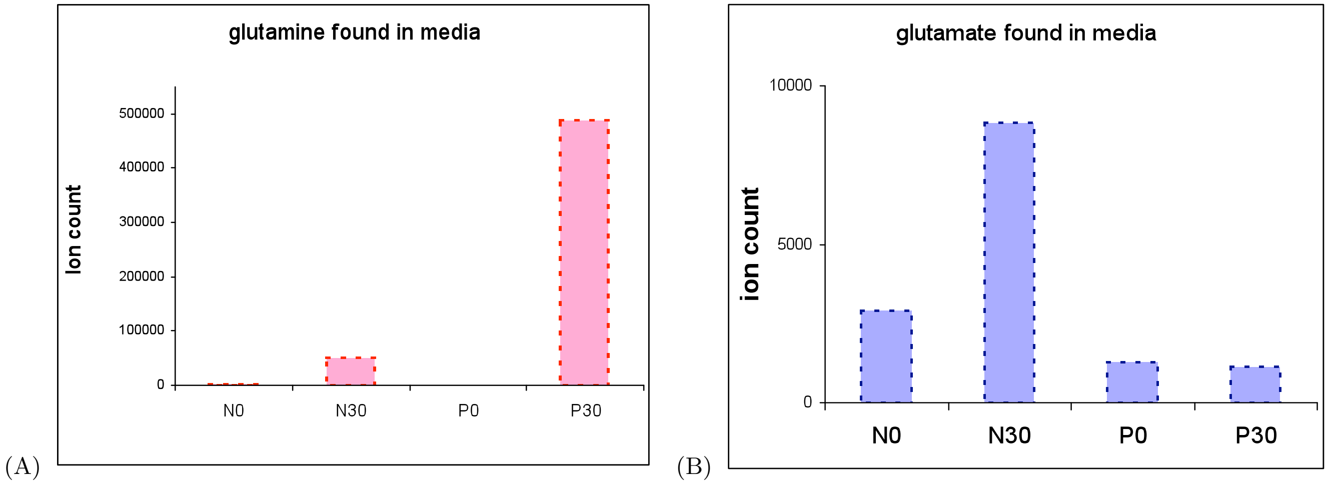

FIG. 3: **Excreted nitrogen intermediates, glutamine and glutamate, in the $\Delta glnE$ (FG 1114) feedback knock-out (KO) strain following a nitrogen upshift. The extracellular nitrogen intermediates are measured for two different nitrogen sources - ammonia (N) and proline (P) at $t = 0$ and $t = 30$ min after the nitrogen upshift is applied. (A) Glutamine found in media. (B) Glutamate found in media.**

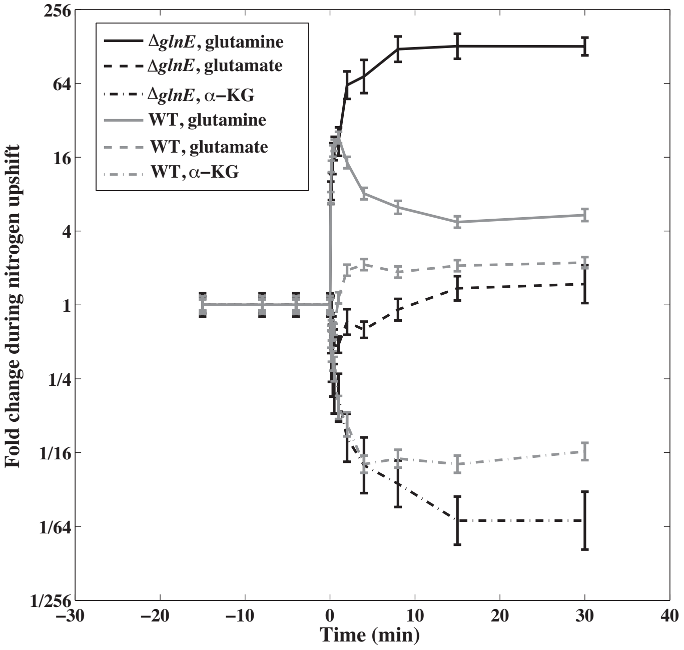

FIG. 4: **Fold changes in key metabolite pools for feedback-knockout Δ*glnE* (FG 1114) and WT strains of *E. coli* under nitrogen upshift. Following the upshift, the trends in the pool size changes of the carbon-intermediate, α-ketoglutarate (α-KG) and the nitrogen intermediates, glutamine and glutamate, are qualitatively similar for the two strains. However, the changes in both α-KG and glutamine are strongly amplified in the feedback-knockout strain (black curves) compared to the WT strain (gray curves).**

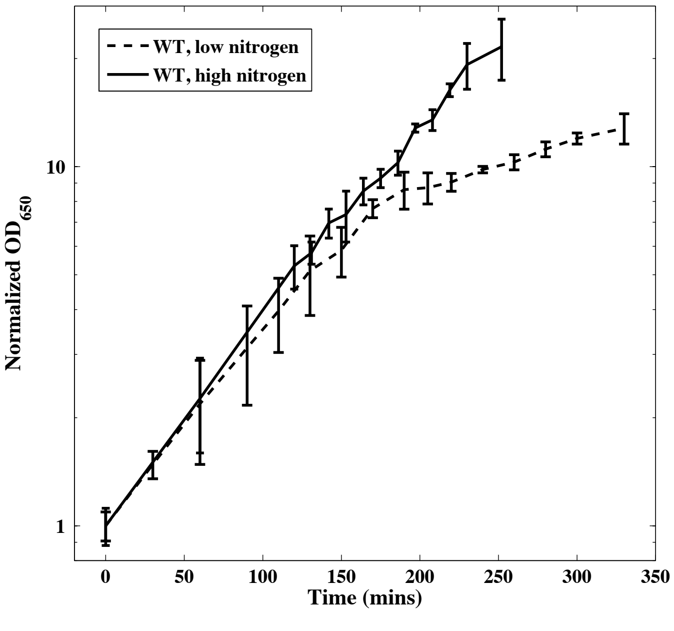

FIG. 5: **Normalized growth curves for WT strain of *E. coli*. At low ammonia concentration (2mM NH_4^+) in the media, the growth rate decreases after $t = 190$ min when the available nitrogen becomes limited (dashed curves). No such decrease is observed in high ammonia concentration (10mM NH_4^+) in the media (solid curve).**
